# Wellens Syndrome: A Possible Precursor

**DOI:** 10.7759/cureus.31963

**Published:** 2022-11-28

**Authors:** Okelue E Okobi, Ibrahim O Bakare, Endurance O Evbayekha, Adedoyin Olawoye, Chioma C Umeh, Amaka Sowemimo

**Affiliations:** 1 Family Medicine, Arizona State University, Tempe, USA; 2 Family Medicine, Lakeside Medical Center, Belle Glade, USA; 3 Internal Medicine, University of Texas Southwestern Medical Center, Dallas, USA; 4 Internal Medicine, St. Luke's Hospital, St. Louis, USA; 5 Internal Medicine, Maimonides Medical Center, New York, USA; 6 Family Medicine, Sure Hope Hospital, Lagos, NGA; 7 General Medicine, University of Maiduguri Teaching Hospital, Maiduguri, NGA

**Keywords:** electrocardiography (ecg), ecg interpretation, critical care cardiology, ekg abnormalities, wellens’ syndrome

## Abstract

Wellens syndrome is a precursor of left anterior descending (LAD) coronary stenosis. It is characterized by biphasic T waves in V2-V3 (type A) or negative deep T waves in V2-V4 (type B). The ability of emergency physicians, hospitalists, or primary care providers to recognize these early ECG patterns is primordial because the definitive treatment is urgent cardiac catheterization with percutaneous coronary intervention. However, failure to identify a type A or type B Wellens syndrome may lead to devastating outcomes, such as myocardial infarction or even death. We presented a clinical case of Wellens’ syndrome with deep T waves in V2-V3 associated with COVID pneumonia, pleural effusions, and congestive heart failure that went to a rapid and massive myocardial infarction.

## Introduction

According to Arisha et al. [1], Wellens syndrome is a type of electrocardiography (ECG pattern of T-waves in the precordial leads), also known as "left anterior descending (LAD) artery T-wave inversion syndrome." The syndrome was first described in the 1980s by Wellens among unstable angina patients with critical LAD coronary stenosis [2]. There are two different types of ECG patterns associated with this syndrome. Type A or "typical Wellens" may present with biphasic T-waves in V2-V3. It is less frequent (25% of cases) but more specific [1-3]. However, type B may show negative T waves in V2-V4. It is more common (75% of cases), but less specific [1-3]. A clinician's ability to recognize these two ECG patterns is crucial because it can help prevent fatal outcomes. The ECG is one of the most valuable tools in the early identification of cardiac disease. Its interpretation requires knowledge, careful attention, experience, and subtle details that can carry immense diagnostic weight. Busy physicians will often abdicate responsibility for ECG interpretation to the cardiologist; however, as the first point of contact for many patients, family physicians must maintain expertise in this area. In many cases, a condition that may be identified on an ECG requires immediate intervention, meaning that consulting a cardiologist may delay care in ways that could produce long-term complications and mortality. Our case described a patient with a deep T-wave inversion pattern in V2-V3 (type B) that nearly went unnoticed if not for the careful eyes of an emergency room physician.

## Case presentation

A 69-year-old male Caucasian with a past medical history of hypertension, diabetes, asthma-chronic obstructive pulmonary disease (COPD), hyperlipidemia, and benign prostatic hyperplasia presented to the emergency department of a small, rural hospital with the chief complaints of dyspnea, orthopnea, and bilateral lower extremity swelling for three weeks. His shortness of breath is alleviated by sitting up and aggravated by supination. Other symptoms include a non-productive cough. However, he denied having chest pain, headache, nausea, or vomiting. His physical examination was within normal limits other than tachypnea (RR: 22), saturation at 96% on room air, and decreased lung breath sounds. On the evaluation of the cardiovascular system, there were no apparent splinter hemorrhages or clubbing, Osler or Jane wane nodules. The extremities were warm and moist; capillary refill time was around four seconds, and there were palpable radial pulses with a regular rate and rhythm. There was no radio-radial or radio-femoral delay. His blood pressure reading was 165/87 mmHg. The jugular venous pressure was normal, and carotid pulses were present on both sides. There were no noticeable scars or visible cardiac impulses on examination of the precordium. The apex beat was normal and located at the fifth intercostal space at the midclavicular line. S1 and S2 heart sounds were normal. Auscultation over the four-valve locations was inconclusive. Murmurs were not appreciated. On lung-base auscultation, there were also fine inspiratory crackles. Pedal edema was prevalent up to the ankle level.

**Figure 1:**
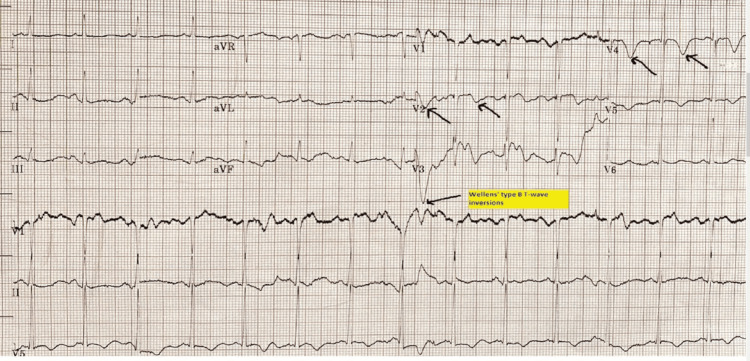
Deep T-wave inversions in V3-V4 in the first EKG series EKG: electrocardiogram. Arrows depicting deep T-wave inversion pattern in V2 and V3 but no ST-elevation myocardial infarction (STEMI) pattern.

In the emergency department, the patient's rapid COVID test was positive. Some biological markers, such as potassium (2.8) and hemoglobin (12.2), were low. Other parameters were elevated, including serum glucose (156), pro-BNP (15703), lactate (2.1), and troponin I (0.164). However, his inflammatory markers, D-dimer, lactate dehydrogenase, ferritin, and C-reactive protein (CRP), were within the standard limit. The chest X-ray showed prominent interstitial pulmonary edema, a small bilateral pleural effusion, and cardiomegaly suggestive of congestive heart failure (CHF). He was treated with Rocephin and Azithromycin for pneumonia, potassium replacement (60 mEq of KCl), and a bolus of 500 mL of normal sodium chloride. His ECG, as represented in Figure 1, showed a deep T-wave inversion pattern in V2 and V3, but no ST-elevation myocardial infarction (STEMI) pattern.

Given the lack of traditional ACS symptoms and the patient's COVID pneumonia, which offered a complicating alternative explanation for his clinical complaints, the cardiology team recommended following the ECGs and troponins expectantly rather than pursuing an immediate transfer.

**Figure 2:**
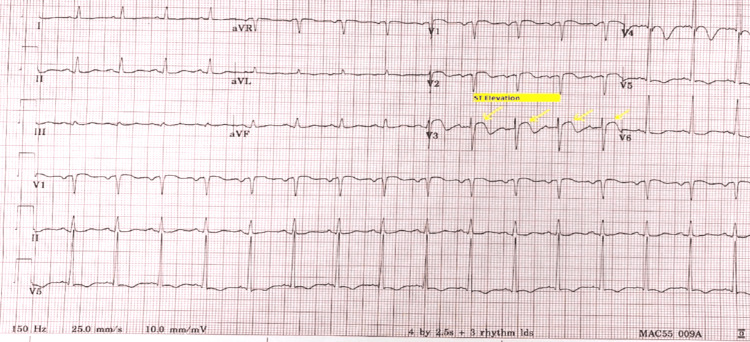
Follow-up EKG with ST elevations EKG: electrocardiogram. Yellow arrows show ST-segment elevations in V3.

The patient was admitted for COVID pneumonia, elevated troponin, and CHF. When the admitting provider rechecked the patient two hours later, he was diaphoretic, dyspneic, and had an O_2_ saturation of 91%. He continued to deny having chest pain. He was placed on BiPAP and, when his symptoms worsened, was transferred to the ICU and intubated. A repeat ECG then revealed ST elevations in V3, V4, and V5, as seen in Figure 2.

**Figure 3:**
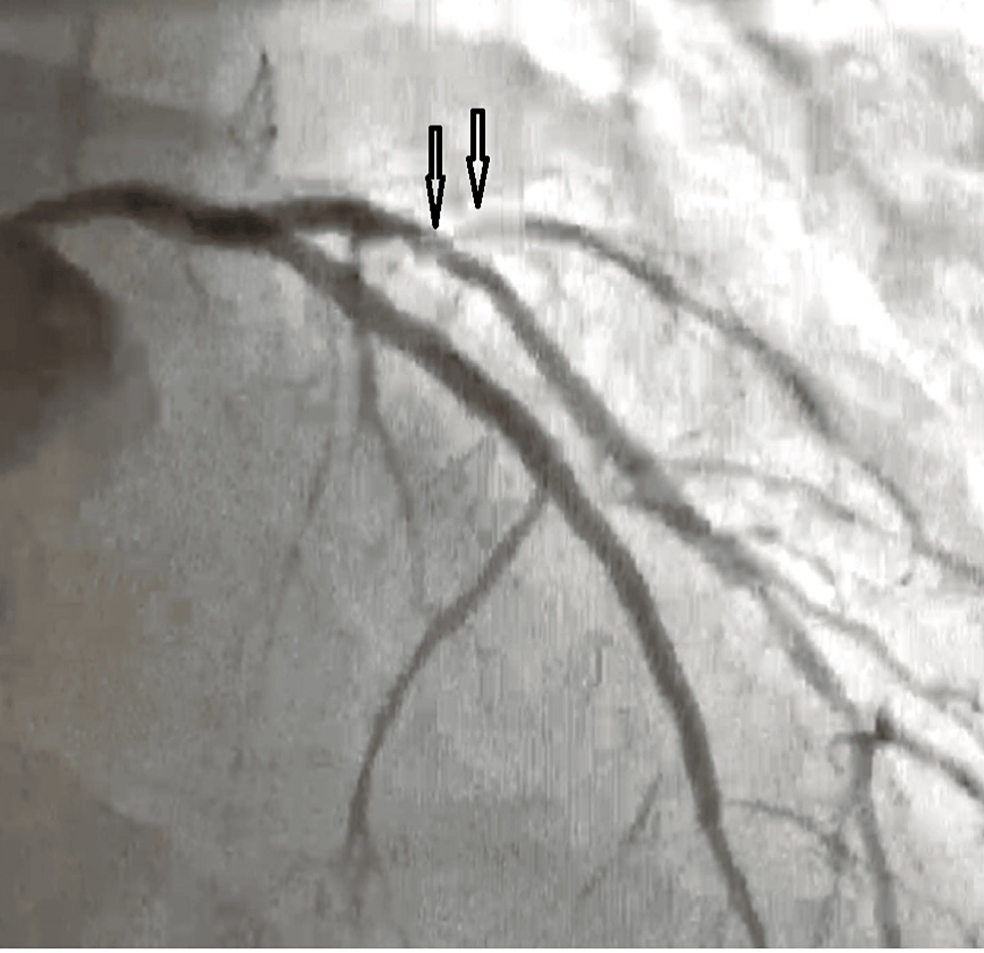
Angiography post-Wellen's syndrome showing LAD occlusion LA: left znterior descending artery.

At that point, the cardiology team recommended initiating an ST-segment myocardial infarction (STEMI) protocol and an urgent transfer for catheterization, though the patient's COVID-19 status complicated the process. Ultimately, this patient's catheterization revealed a proximal occlusion of the LAD artery, as seen in Figure 3.

The patient's post-catheterization condition was acceptable, and he was discharged for follow-up. However, the patient did not return to his main rural hospital for a check-up; thus, the exact details of his outpatient vitals and condition could not be ascertained.

## Discussion

Wellens syndrome has the same risk factors as traditional acute coronary syndrome, including HTN, diabetes, hyperlipidemia, smoking, etc. [1-4]. Like previously recorded examples of Wellen's syndrome in several classic documentations [5-9], our patient had a history of high blood pressure, diabetes, and hyperlipidemia, which predisposed him to this pre-infarction condition, comparable to those that cause coronary artery disease. Postulations have been made that the alteration in the EKG is accounted for by the reperfusion of the ischemic myocardium owing to the reduction of spasm in the proximal LAD artery. Still, the cause of Wellens' syndrome remains unclear [5-9]. Furthermore, these ECG changes representing a pre-infarction state are commonly associated with unstable angina [1-4]. Also, the exact electrophysiologic mechanism is unknown [1-4]. While the exact electrophysiologic mechanism is unknown, the Wellens syndrome pattern may be evidence of temporary occlusion of the LAD coronary artery [2-4]. Plaque rupture could quickly produce a complete LAD obstruction and MI. This patient's rapid development of diaphoresis and dyspnea likely represented exactly this conversion.

Usually, the assessment and diagnosis include a triad: primarily, ECGs follow one of two patterns (type A with biphasic T-waves in V2-V3 or type B with negative T waves in V2-V4) with a minimally elevated or isoelectric ST-segment in a patient who often has no acute pain. Compared with documented cases of approximately one-quarter of these patients having these changes in V1 and two-thirds having these changes in V4 [5-8], our patient presented with classic wave patterns in V2-V4. In a Chinese study that looked back at 3528 individuals who had angioplasty between 2017 and 2019, 5.7% (200) of them fit the criteria for Wellen's syndrome using this EKG pattern [9]. Second, cardiac enzymes are in the normal or near-normal range. Finally, patients have a history of angina or other markers of coronary artery disease [1-4].

The patient presented pain-free with a slight elevation of cardiac enzymes and the context of a severe alternative diagnosis. The absence of pain or definitive evidence of acute infarction prevented the primary team from pressing for the immediate aggressive interventions they would have if they were confident of this patient's acute coronary syndrome. This ominous clinical presentation has been seen in numerous previous known instances of Wellen's syndrome. Because of this, every clinician needs to understand these patterns. This can help them make better clinical decisions and lead to more aggressive responses, lowering deaths related to the problem.

A multidisciplinary management approach has been documented to improve patient outcomes in various clinical settings [1-7,9], with the involvement of early interventional cardiology. Some guidelines [9-11] support cardiac catheterization in conjunction with percutaneous coronary intervention. Until then, evidence-based good clinical judgment requires the physicians to treat Wellen's syndrome as if it were an acute myocardial infarction, with heparin for anticoagulation and antiplatelet medications like aspirin, nitrates, and beta blockers for non-hypotensive patients. Similarly, keeping with these recommendations and historically documented cases, the patient was managed in line with these recommendations. Although COVID-19 was a significant confounder in deciding his management protocol, during admission, our patient received ceftriaxone, azithromycin, and dexamethasone for his COVID pneumonia and furosemide and atenolol for his CHF and hypertension. If Wellen's pattern arose from vasospasm rather than atherosclerosis, calcium channel blockers or nitroglycerin would be recommended, though they were not included in this patient's care [1-4].

Early percutaneous coronary intervention is the preferred treatment modality [1-3,6-10] for NSTE acute coronary syndrome, especially in pain-free patients like this patient. Still, in an ideal world and at the peak of the COVID-19 era, few patients received such prompt percutaneous intervention. Post-catheterization outcomes have also been very successful for those who received these quick interventions, but a delay in intervention may lead to anterior wall infarction and, in some cases, death [5-9,12].

## Conclusions

Wellens syndrome is a premonitory sign of MI. Hospitalists and emergency physicians must be aware of these EKG patterns. Unfortunately, 75% of patients with these EKG findings are at significant risk for MI within weeks if treated only with medical management. Early recognition of these ECG features is crucial to identify these high-risk clients, and the definitive evaluation and treatment is cardiac catheterization with intervention to relieve the LAD obstruction. In those cases, when the diagnosis is inconclusive and the treatment team elects to manage medically, the team should be vigilant to the potential for rapid deterioration.
